# LncRNA GHET1 promotes cervical cancer progression through regulating AKT/mTOR and Wnt/β-catenin signaling pathways

**DOI:** 10.1042/BSR20191265

**Published:** 2020-01-03

**Authors:** Zhihui Liu, Sukun Luo, Meiqin Wu, Chong Huang, Huifen Shi, Xiaojie Song

**Affiliations:** 1 Department of Gynecology, Wuhan Children’s Hospital (Wuhan Maternal and Child Healthcare Hospital), Tongji Medical College, Huazhong University of Science and Technology, Wuhan 430016, Hubei, China; 2 Precision Medical Laboratory, Wuhan Children’s Hospital (Wuhan Maternal and Child Healthcare Hospital), Tongji Medical College, Huazhong University of Science and Technology, Wuhan 430016, Hubei, China; 3 Department of Gynecology, The Second People’s Hospital of Nanhai District, Foshan, Guangdong, China

**Keywords:** AKT/mTOR, cervical cancer, GHET1, Wnt/β-catenin

## Abstract

Cervical cancer (CC) is a prevalent gynecological cancer, and the patients with CC usually suffer from dismal prognosis. Long non-coding RNAs (lncRNAs) are demonstrated to serve as promising biological targets in human cancers. Gastric carcinoma proliferation enhancing transcript 1 (GHET1) has been revealed to function as an oncogene in several cancers, but it has never been investigated in CC. We proposed to examine the biological role of GHET1 in CC and the underlying mechanism and validated the up-regulated expression of GHET1 in CC cell lines. Loss-of-function assays demonstrated that down-regulation of GHET1 inhibited cell growth, migration and epithelial-to-mesenchymal transition (EMT) in CC. Furthermore, we validated that GHET1 down-regulation could inactivate AKT/mTOR and Wnt/β-catenin pathways, and that respective activation of these two pathways abrogated the inhibitive effect of GHET1 knockdown on CC cell growth, migration and EMT. Moreover, we unfolded a preliminary investigation on the modulation of GHET1 on AKT/mTOR and Wnt/β-catenin pathways. We found that GHET1 stabilized E2F6 mRNA through interacting with IGF2BP2, so as to regulate the activity of AKT/mTOR and Wnt/β-catenin pathways. Rescue assays also proved that GHET1 regulated these two pathways and CC cell growth, migration and EMT through E2F6. In conclusion, we revealed that down-regulation of GHET1 suppresses cervical cancer progression through regulating AKT/mTOR and Wnt/β-catenin signaling pathways, indicating GHET1 as a promising molecular biomarker for CC treatment improvement.

## Introduction

As a familiar gynecological cancer, cervical cancer (CC) accounts for a large proportion of tumor-resulted deaths globally [1,2]. Although there are various therapeutic regimes accessible to CC patients, including tumor dissection and chemo- or radiotherapies, only approximately 40% of CC patients could survive for more than 5-years [3,4]. Therefore, further molecular research is required to be done in order to identify promising biological targets and improve the prognosis in CC.

Long non-coding RNAs (lncRNAs), a set of transcribed RNAs with over 200 nucleotides in length, are incapable of translating proteins [5]. Mounting studies have demonstrated that dysregulated lncRNAs have close relation to tumorigenesis and progression of human cancers [6,7], including CC [8]. Gastric carcinoma proliferation enhancing transcript 1 (GHET1) is a lncRNA suggested by several studies as an oncogene in cancers, such as breast cancer [9], colorectal cancer [10] and gastric cancer [11]. However, the role of GHET1 has not been explored in cervical cancer.

The tumor development involves the activation and inactivation of multiple pathways. AKT/mTOR is an essential signaling pathway responsible for the modulation of cancer cell biological activities such as proliferation and migration [12]. The activation of AKT/mTOR involves phosphorylation of AKT and mTOR. Interestingly, studies have shown that activated AKT could result in phosphorylation and inactivation of GSK3β [13,14], which therefore promotes β-catenin accumulation and nuclear translocation in Wnt/β-catenin pathway [15]. These previous findings indicate the crosstalk between AKT/mTOR and Wnt/β-catenin signaling pathways, and the regulation of these two pathways has also been reported in several cancers [16,17]. Although both AKT/mTOR and Wnt/β-catenin pathways have been investigated in cervical cancer respectively [18,19], no studies have been reported on the regulation of these pathways by GHET1 in CC.

E2F transcription factor 6 (E2F6) belongs to the family of E2F transcription factors that regulate gene transcription and influence cell differentiation, proliferation, cell cycle and apoptosis [20]. Multiple studies have revealed the up-regulation and oncogenic function of E2F6 in cancers, such as ovarian cancer and endometrial carcinoma [21,22]. Importantly, it has been proved that E2F6 could regulate both AKT/mTOR and Wnt/β-catenin pathways in Ewing’s sarcoma [17]. However, the function of E2F6 in CC tumor progression and its relation with GHET1 have never been explored.

Present study planned to investigate the function of GHET1 in cervical cancer and its mechanism in regulating AKT/mTOR and Wnt/β-catenin signaling pathways.

## Materials and methods

### Tissue samples

Forty patients from Wuhan Children’s Hospital (Wuhan Maternal and Child Healthcare Hospital), Tongji Medical College, Huazhong University of Science and Technology were participated in the present study. None of them experienced chemo- or radiation therapy. After surgical resection, tumor tissues were kept in liquid nitrogen at once and were reserved under −80°C for following use. This research was approved by the Ethics Committee of Wuhan Children’s Hospital (Wuhan Maternal and Child Healthcare Hospital), Tongji Medical College, Huazhong University of Science and Technology. Informed consent was attained from all the patients.

### Cell lines and cell culture

Four human CC cell lines (C33A, HeLa, C4-1 and SiHa) and the normal cervical cell line (Crl-2614) were provided by American Type Culture Collection (Manassas, VA, U.S.A.). The culture medium of all cell lines was Dulbecco’s Modified Eagle’s Medium (DMEM; Thermo Fisher Scientific, Waltham, MA, U.S.A.). Media were with the supplementation of 10% fetal bovine serum (FBS) and 1% penicillin/streptomycin (all from Gibco; Thermo Fisher Scientific, Inc.), and were maintained at 37°C in a humidified incubator with 5% CO_2_. Cells that were exponentially growing were collected for later experiments.

### Cell transfection

GHET1/IGF2BP2 was knocked down by two specific small interfering RNAs (siRNAs) targeting GHET1 (siGHET1#1/siIGF2BP2#1 and siGHET1#2/siIGF2BP2#2), with the scramble siRNA (GenePharma, Shanghai, China) as negative control. IGF2BP2 and E2F6 were overexpressed by inserting the sequences of IGF2BP2 or E2F6 into pcDNA3.1, with vector pcDNA3.1 (Thermo Fisher Scientific, Inc., Waltham, MA, U.S.A.) acting as negative control. The transfection of these plasmids into CC cells was conducted by Lipofectamine 2000 (Invitrogen, Carlsbad, CA, U.S.A.).

### Quantitative real-time PCR (qRT-PCR)

The RNA extracts were obtained from the harvested CC cells by the use of TRIzol® reagent (Invitrogen; Thermo Fisher Scientific, Inc., Waltham, MA, U.S.A.), and the RNase-Free DNase Set (Qiagen, Inc., Valencia, CA, U.S.A.) was used to purify the RNAs. Subsequently, purified RNAs were subjected to the reverse transcription into complementary DNA (cDNA) with the use of SuperScript III Reverse Transcriptase (Invitrogen; Thermo Fisher Scientific, Inc.). Then, qPCR was implemented on the ABI 7300 Real-Time PCR System (Applied Biosystems; Thermo Fisher Scientific, Inc.) by the application of Power SYBR Green Master Mix Kit (Thermo Fisher Scientific Inc, Waltham, MA, U.S.A.). The calculation of gene expressions was conducted on the basis of the 2^−ΔΔ*C*^_t_ method. The internal control was GAPDH. The primer sequences are presented in Table 1.

**Table 1:** Primer sequences

GHET1	5′ TACCACACCCTTTCTTGCCC 3′ (forward),
	5′ GGGAGCCAAAAGGGTCA 3′ (reverse);
E2F6	5′ GACCTCGTTTTGATGTATCGCTG (forward),
	5′ ATACACTCTCCGCTTTCGGAC (reverse);
IGF2BP2	5′ ACCCTCTCGGGTAAAGTGGA 3′ (forward),
	5′ GTTGACAACGGCGGTTTCTG (reverse);
GAPDH	5′ TCGGAGTCAACGGATTTGGT 3′ (forward),
	5′ TTCCCGTTCTCAGCCTTGAC 3′ (reverse).

### Cell counting kit-8 (CCK-8) assay

The proliferation of CC cells was examined applying the Cell Counting Kit-8 (CCK-8, Dojindo, Kumamoto, Japan) reagent. In short, CC cells were plated into the 96-well plates (1 × 10^3^/well). At 0, 24, 48, 72 and 96 h after incubation respectively, each well was added with CCK-8 solution (10 μl) followed by the incubation of CC cells at 37°C for 2 h. Measurement of the absorbance (wavelength: 450 nm) in each well was carried out applying a microplate absorbance reader (Bio-Rad Laboratories, Hercules, CA).

### Transwell migration

In the preparation for migration assay, CC cells were cultured in non-serum media 2 days after transfection and were placed in higher chamber of the insert (8-μm pore size, Millipore, Billerica, MA, U.S.A.) for migration assay. The media with 10% FBS was added to lower chamber. After that, CC cells that had migrated through the membrane were subjected to the staining in methanol with 0.1% Crystal Violet. Then, IX71 inverted microscope (Olympus, Tokyo, Japan) was applied to observe and count cells.

### TOP-Flash assay

The construction of β-catenin reporter plasmid (TOP-flash) as well as the mutant control (FOP-flash) was accomplished by Millipore Corporation (Massachusetts, U.S.A.). Then, TOP-flash or the FOP flash expression plasmids were co-transfected with pRL-TK (Renilla TK-luciferase vector; Promega, Madison, U.S.A.) into CC cells that were previously serum-starved for 12 h by means of Lipofectamine 2000 (Invitrogen). Determination of firefly or Renilla luciferase activities was carried out at 48 h following transfection applying a Dual Luciferase Assay Kit (Promega, Madison, WI, U.S.A.). The activity of TOP-Flash reporter was presented as the relative proportion of firefly luciferase activity to the Renilla luciferase activity. TOP/FOP ratio was utilized to examine the β-catenin-driven transcription.

### Immunofluorescence staining (IF)

After the 1 h incubation of CC cells with blocking buffer (5% normal goat serum, 3% bovine serum albumin and 0.1% Triton-X 100 in PBS), the primary antibodies against β-catenin (Abcam, Cambridge, U.K.) were added and incubated with the CC cells overnight. Following rinsing for three times with PBST, cells were then incubated with secondary antibodies for 1 h under room temperature. DAPI (Burlingame, CA) was then used to stain the cell nuclei. Pictures were taken applying the Nikon Ti inverted fluorescence microscope.

### mRNA stability assay

After the transfection for 48 h, the Actinomycin D (ActD) (5 μg/ml, Apexbio, U.S.A.) was used to inhibit *de novo* RNA. Following the harvest of total RNA at indicated time points, the expression of E2F6 mRNA was evaluated by qRT-PCR. The half-life of E2F6 mRNA was examined by comparing the mRNA expression of E2F6 to that of E2F6 before adding ActD.

### RNA immunoprecipitation (RIP)

Magna RIP RNA-Binding Protein Immunoprecipitation Kit (Millipore, Stafford, VA) was applied for RIP assay. After CC cells were lysed in the complete RIP lysis buffer, the whole cell extracts were subjected to the overnight incubation with RIP buffer magnetic beads with antibodies against IGF2BP2 (Abcam, Cambridge, U.K.) at 4°C, with IgG (Abcam) as negative control. Then, the purified RNAs in the precipitates were evaluated by RT-qPCR.

### Western blot

After being lysed with RIPA Lysis Buffer (Beyotime, Beijing, China), the protein density of CC cells was examined using Bradford Protein Assay Kit (Beyotime). Subsequently, proteins were subjected to the separation by 10% sodium dodecyl sulfate-polyacrylamide gel (SDS-PAGE). Then, proteins were transferred onto the polyvinylidene difluoride (PVDF) membranes (Bio-Rad, Hercules, CA, U.S.A.), followed by the blocking in 10% non-fat milk at 37°C for 1.5 h. Thereafter, membranes were washed and detected with the primary antibodies for 12 h at 4°C and were subsequently incubated with the secondary antibodies for 2 h. Examination of protein bands was carried out utilizing the enhanced chemiluminescence with imaging system (Bio-Rad). The primary antibodies against E-cadherin, N-cadherin, p-AKT, AKT, p-mTOR, mTOR, p-GSK3β, total p-GSK3β, β-catenin, Cyclin D1, c-Myc, E2F6 and GAPDH were purchased from Abcam (Cambridge, U.K.).

### Statistical analysis

All assays were conducted ≥3 times. The data presentation was carried out as mean ± standard deviation. Data analysis was carried out utilizing SPSS 16.0 software (SPSS, Inc., Chicago, IL, U.S.A.). The determination of statistical differences between two groups or among multiple groups was performed utilizing the Student’s *t*-test or one-way ANOVA. *P* < 0.05 suggested significance at a statistical level.

## Results

### GHET1 was up-regulated in CC cells and its down-regulation inhibited proliferation, migration and EMT

**Figure 1:**
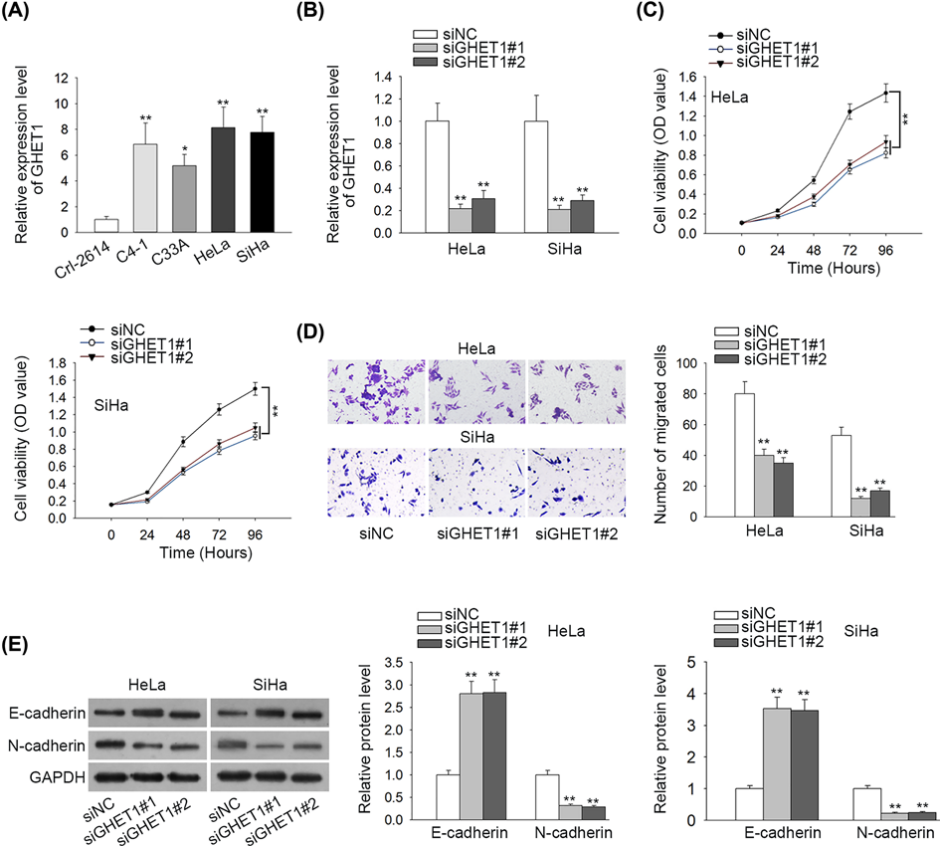
GHET1 down-regulation inhibited CC cell growth, migration and EMT (**A**) RT-qPCR revealed GHET1 expression in CC cells and normal cells. (**B**) GHET1 was silenced by siRNAs and the knockdown was determined by RT-qPCR analysis. (**C**) Proliferation of HeLa and SiHa cells upon GHET1 down-regulation was determined by CCK-8 assay. (**D**) Migration of HeLa and SiHa cells upon GHET1 down-regulation was determined by Transwell migration assay. (**E**) Western blot was applied to detect expressions of E-cadherin and N-cadherin; *P < 0.05, **P < 0.01.

We first investigated how GHET1 affected the biological activities of CC cells. As shown in Figure 1A, GHET1 presented elevated expression in CC cell lines (C4-1, C33A, HeLa and SiHa) compared with normal cell line (Crl-2614). Since HeLa and SiHa cells presented the highest level of GHET1, they were used for the following experiments. Next, we knocked down GHET1 in HeLa and SiHa cells for function assays. The expression of GHET1 was confirmed to decrease in two CC cell lines transfected with siGHET1#1 or siGHET1#2 (Figure 1B). After that, we observed through CCK-8 assay that knocking down GHET1 prohibited CC cell proliferation (Figure 1C). Transwell migration assay validated that down-regulation of GHET1 decreased migratory ability of CC cells (Figure 1D). Additionally, E-cadherin (epithelial marker) expression was enhanced, whereas N-cadherin expression (mesenchymal marker) was decreased upon GHET1 knockdown in CC cells (Figure 1E), indicating that GHET1 suppression might inhibit EMT progression in CC cells. Jointly, these results suggested that GHET1 was up-regulated in CC cells and its down-regulation inhibited proliferation, migration and EMT.

### Knockdown of GHET1 suppressed AKT/mTOR and Wnt/β-catenin pathways in CC

**Figure 2:**
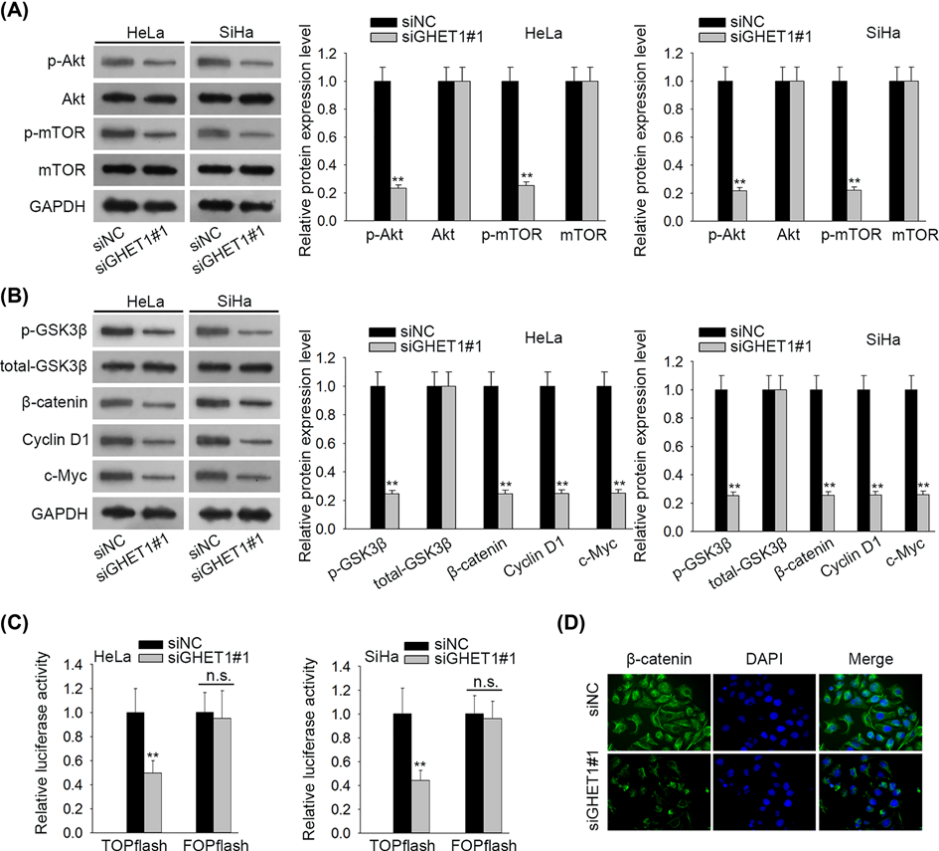
GHET1 down-regulation inhibited AKT/mTOR and Wnt/β-catenin pathways in CC (**A** and **B**) Expressions of proteins in AKT/mTOR and Wnt/β-catenin pathways (total and phosphorylated AKT and mTOR, total and phosphorylated GSK3β, β-catenin, Cyclin D1 and c-Myc) were detected through Western blot analyses. (**C**) Activity of Wnt/β-catenin pathway was examined by TOP-FOP flash assay. (**D**) IF staining was used to examine β-catenin expression; **P < 0.01.

It has been acknowledged that AKT/mTOR pathway performs essential part in regulating cancer cell biological activities such as proliferation and migration [12], including in CC [23]. Therefore, we speculated whether GHET1 exerted its influence through regulating AKT/mTOR pathway. Results of Western blot showed that down-regulating GHET1 reduced expression of p-AKT and p-mTOR in HeLa and SiHa cells, but had no effect on total AKT and mTOR (Figure 2A), indicating that GHET1 positively regulated AKT/mTOR pathway. Moreover, it has been proved that p-AKT could induce the phosphorylation GSK3β [13,14], which therefore leads to acumulation and nuclear migration of β-catenin [15]. Hence, we probed effects of GHET1 on Wnt/β-catenin pathway. As a result, knockdown of GHET1 decreased the levels of p-GSK3β, β-catenin and downstream effectors including c-Myc and Cyclin D1, but didn’t affect the level of total GSK3β (Figure 2B). Also, TOP-flash assay validated that silencing GHET1 hindered Wnt/β-catenin pathway activity in HeLa and SiHa cells (Figure 2C). Furthermore, IF staining assay showed that β-catenin expression in both cytoplasm and nuclear declined in response to GHET1 depletion in HeLa cells (Figure 2D). Altogether, these results indicated that knockdown of GHET1 inhibited AKT/mTOR and Wnt/β-catenin pathways in CC.

### GHET1 regulated cell proliferation and migration through AKT/mTOR and Wnt/β-catenin pathways

**Figure 3:**
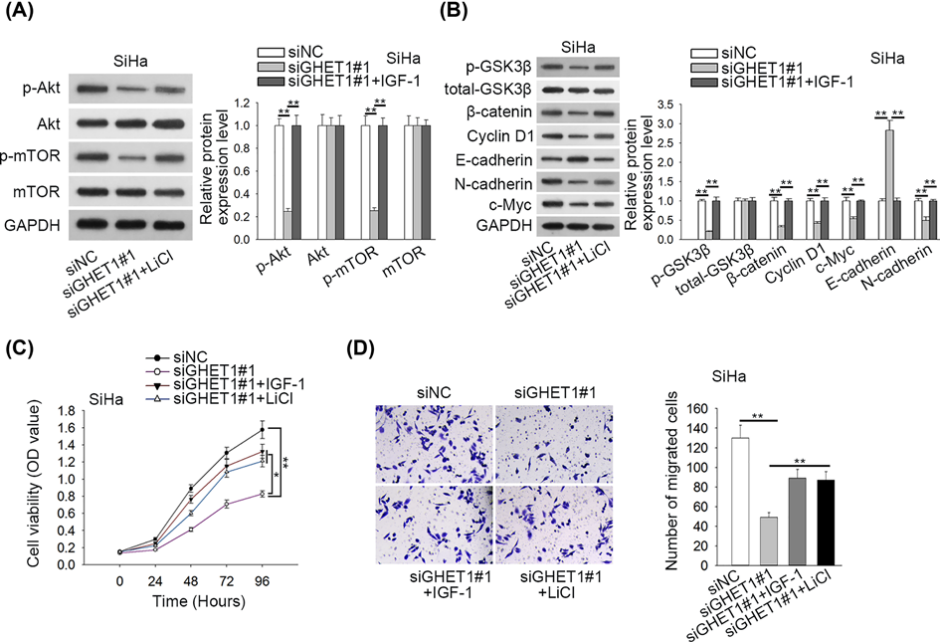
GHET1 regulated cell proliferation and migration through AKT/mTOR and Wnt/β-catenin pathways SiHa cells were treated with siNC, siGHET1#1, siGHET1#1+IGF-1 or siGHET1#1+LiCl for subsequent assays. (**A** and **B**) The proteins in AKT/mTOR and Wnt/β-catenin pathways as well E-cadherin and N-cadherin were detected by Western blot analyses. (**C** and **D**) CCK-8 and transwell migration assays were used to evaluate proliferation and migration of SiHa cells; *P < 0.05, **P < 0.01.

Then, rescue assays were implemented to examine whether GHET1 regulated CC cell proliferation and migration through AKT/mTOR and Wnt/β-catenin patterns. We found that the treatment of IGF-1 (activator of AKT/mTOR pathway) impaired repressive effect of siGHET1#1 on levels of p-AKT and p-mTOR in SiHa cells (Figure 3A). Also, treatment of LiCl (the activator of Wnt/β-catenin pathways) restored decreased p-GSK3β, β-catenin, c-Myc, Cyclin D1, N-cadherin expressions and reversed increased E-cadherin expression in SiHa cells with GHET1 knockdown, and level of total GSK3β wasn’t affected (Figure 3B). Further, we validated that treatment of either IGF-1 or LiCl could impair repressive influence of GHET1 depletion on CC cell proliferation and migration (Figure 3C,D). Hence, these data suggested that GHET1 regulated cell proliferation and migration through AKT/mTOR and Wnt/β-catenin pathways.

### GHET1 regulated AKT/mTOR and Wnt/β-catenin pathways and CC progression through interacting with IGF2BP2 to stabilize E2F6

**Figure 4:**
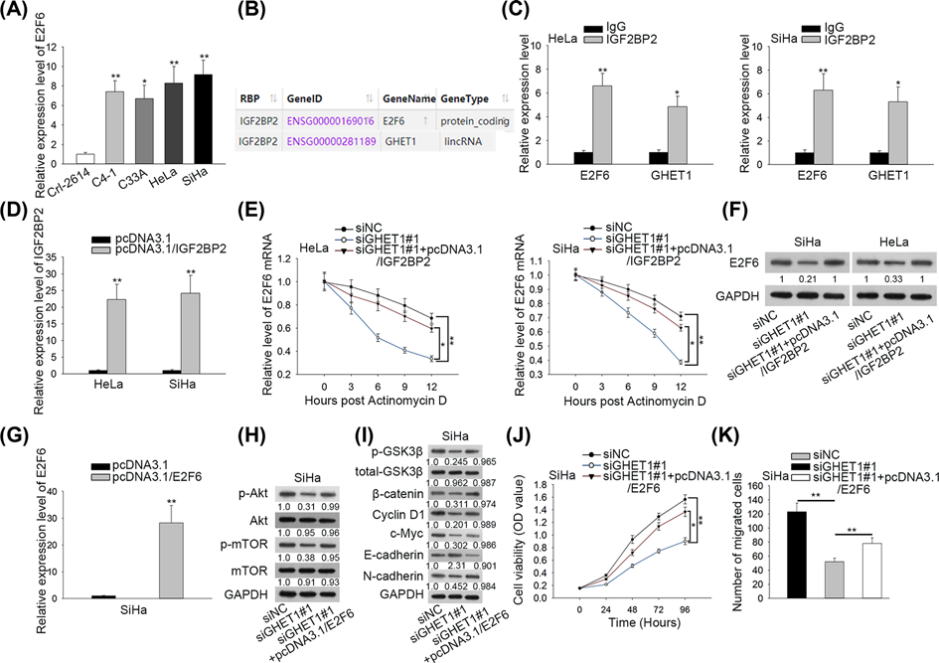
GHET1 regulated AKT/mTOR and Wnt/β-catenin pathways and CC progression through interacting with IGF2BP2 to stabilize E2F6 (**A**) Expression of E2F6 in normal and CC cell lines was evaluated by RT-qPCR. (**B**) The interaction of IGF2BP2 with GHET1 and E2F6 was obtained from Starbase. (**C**) The interaction of IGF2BP2 with GHET1 and E2F6 was confirmed by RIP assay. (**D**) Overexpression of IGF2BP2 in HeLa and SiHa cells was determined by RT-qPCR. (**E**) The half-life of E2F6 mRNA was determined by RT-qPCR. (**F**) Expression of E2F6 was determined by Western blot. (**G**) Overexpression of E2F6 in SiHa cells was determined by RT-qPCR. (**H** and **I**) The proteins in AKT/mTOR and Wnt/β-catenin pathways as well E-cadherin and N-cadherin were detected by Western blot analyses. (**J** and **K**) SiHa cell proliferation and migration were detected by CCK-8 and transwell assays; *P < 0.05, **P < 0.01.

Finally, we sought to investigate how GHET1 regulated AKT/mTOR and Wnt/β-catenin pathways. E2F6 could regulate AKT/mTOR and Wnt/β-catenin pathways in Ewing’s sarcoma [17]. Also, E2F6 was an oncogene in multiple cancers such as endometrial carcinoma [21,22]. So we speculated that GHET1 might be able to regulate E2F6 to regulate AKT/mTOR and Wnt/β-catenin patterns in CC. E2F6 expression was first detected in cells, and it was disclosed that E2F6 was up-regulated in CC cell lines in contrast with normal cells, (Figure 4A). Previous study has demonstrated in gastric cancer that GHET1 could interact with IGF2BP1, a member of IGF-2 binding proteins, to stabilize c-Myc mRNA [24]. Interestingly, we found through Starbase (http://starbase.sysu.edu.cn/index.php) that IGF2BP2, another member of IGF-2 binding protein family, was a RNA-binding protein potentially interacting with both GHET1 and E2F6 (Figure 4B). IGF2BP2 has been reported to stabilize multiple mRNAs and promoted embryonic rhabdomyosarcoma [25]. Therefore, we hypothesized that GHET1 might regulate E2F6 expression through IGF2BP2. RIP assays confirmed that both GHET1 and E2F6 mRNA were enriched in the precipitates of anti-IGF2BP2 (Figure 4C). Subsequently, we overexpressed IGF2BP2 in CC cells (Figure 4D). Besides, the knockdown efficiency of IGF2BP2 was detected in siIGF2BP2#1/siIGF2BP2#2 transfected cells (Supplementary Figure S1A). And as shown in Supplementary Figure S1B and C, siIGF2BP2#1 decreased the expression and protein level of E2F6, while the expression of lncRNA GHET1 wasn’t affected. After treating HeLa and SiHa cells with Actinomycin D, we detected the mRNA stability of E2F6 by examining its mRNA levels over time. Consequently, silencing GHET1 shortened the half-life of E2F6 mRNA, but such effect could be countervailed by overexpressing IGF2BP2 (Figure 4E). Western blot analysis demonstrated that E2F6 protein expression declined upon GHET1 down-regulation, which could be restored by overexpression of IGF2BP2 (Figure 4F). In addition, siIGF2BP2#1 blocked AKT/mTOR and Wnt/β-catenin pathway (Supplementary Figure S1D and E). These results indicated that GHET1 regulated AKT/mTOR and Wnt/β-catenin pathways through recruiting IGF2BP2 to stabilize E2F6. More importantly, the levels of GHET1, IGF2BP2 and E2F6 were assessed in CC tissues and normal tissues. As a result, GHET1, IGF2BP2 and E2F6 presented elevated expression in CC tissues compared with non-tumor tissues (Supplementary Figure S2A–C).

Finally, we probed whether GHET1 regulated CC progression through E2F6. We overexpressed E2F6 in SiHa cells (Figure 4G). Overexpression of E2F6 countervailed the inhibitory effect of GHET1 down-regulation on AKT/mTOR and Wnt/β-catenin pathways (Figure 4H,I). Besides, the co-transfection of pcDNA3.1/E2F6 could offset the restrained proliferation and migration of SiHa cells caused by GHET1 down-regulation (Figure 4J,K). In sum, these data suggested that GHET1 regulated CC progression through E2F6.

## Discussion

LncRNAs are increasingly illustrated by mounting studies to have significant impact on cancer regulation [6,7], including in cervical cancer [8]. These findings indicate that uncovering new lncRNAs in cervical cancer might be helpful for providing promising therapeutic targets and improve the prognosis for CC patients. Previous studies have shown that lncRNA GHET1 functioned as an oncogene in several cancers [9–11]. Herein, our study first discovered that GHET1 was overexpressed in CC cells. Further, silencing GHET1 exhibited inhibitory effects on proliferation, migration and EMT of CC cells.

Former studies have delineated that AKT/mTOR pathway and Wnt/β-catenin pathway are key pattern regulating cancer progression [12,26], including in cervical cancer [18,19]. Also, several studies have revealed the crosstalk of these two pathways by demonstrating that p-AKT could induce the phosphorylation GSK3β so as to inhibit GSK3β [13,14], which consequently leads to β-catenin accumulation and nuclear migration, thus activating Wnt/β-catenin pathway [15]. Our study first related GHET1 to AKT/mTOR and Wnt/β-catenin pathways in CC by showing that down-regulating GHET1 reduced the activities of AKT/mTOR pathway and Wnt/β-catenin pathway. Furthermore, we confirmed that activating either AKT/mTOR pathway or Wnt/β-catenin pathway could impair GHET1 down-regulation mediated inhibitory influence on proliferation, migration and EMT in CC, indicating that GHET1 regulated CC progression through these two pathways.

Furthermore, we probed the mechanism whereby GHET1 regulated AKT/mTOR pathway and Wnt/β-catenin pathway. A former study has shown that E2F6 could regulate both AKT/mTOR and Wnt/β-catenin pathways in Ewing’s sarcoma [17]. E2F6 belongs to the family of E2F transcription factors, and has been reported to exert oncogenic functions in several gynecological cancers, such as ovarian cancer and endometrial carcinoma [21,22], but the role of E2F6 in CC remains elusive. These findings prompted us to speculate that GHET1 might regulate AKT/mTOR pathway and Wnt/β-catenin pathway through E2F6. We first explored how GHET1 regulated E2F6 in CC. According to previous study, GHET1 interacted with IGF2BP1 to stabilize c-Myc mRNA so as to promote gastric cancer [24]. It has been known that IGF2BP1 is a member of IGF2-binding proteins responsible for the regulation of mRNA stability, translation and localization [27]. In our study, we found through Satrbase that IGF2BP2, another member of IGF2-binding proteins, was a shared interacting partner for GHET1 and E2F6. Previous study has proved that IGF2BP2 could stabilize multiple mRNAs and promoted embryonic rhabdomyosarcoma [25]. However, never has IGF2BP2 been explored in CC. Therefore, the present study was the first to uncover in CC that GHET1 interacted with IGF2BP2 to stabilize E2F6 mRNA. Finally, rescue assays suggested that GHET1 regulated CC progression through E2F6.

To be concluded, our findings first showed that knockdown of GHET1 inhibited proliferation, migration and EMT through AKT/mTOR and Wnt/β-catenin pathways, suggesting GHET1 as a novel biological target in CC and providing new thoughts for the improvement of treatment of CC.

## Supplementary Material

Supplementary Figures S1-S2

## Abbreviations

CC: cervical cancer
EMT: epithelial-to-mesenchymal transition
GHET1: gastric carcinoma proliferation enhancing transcript 1
IF: immunofluorescence staining
LncRNA: long non-coding RNA
RIP: RNA immunoprecipitation
