# Supplementary figures and images for: LncRNA GHET1 promotes cervical cancer progression through regulating AKT/mTOR and Wnt/β-catenin signaling pathways
Source: Biosci Rep. 2020 Jan 3;40(1):BSR20191265. doi: 10.1042/BSR20191265 (PMC6944656; doi:10.1042/BSR20191265)

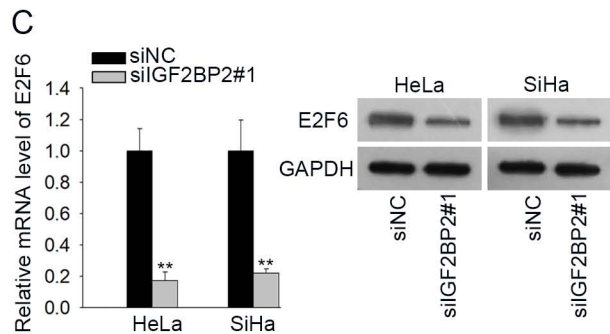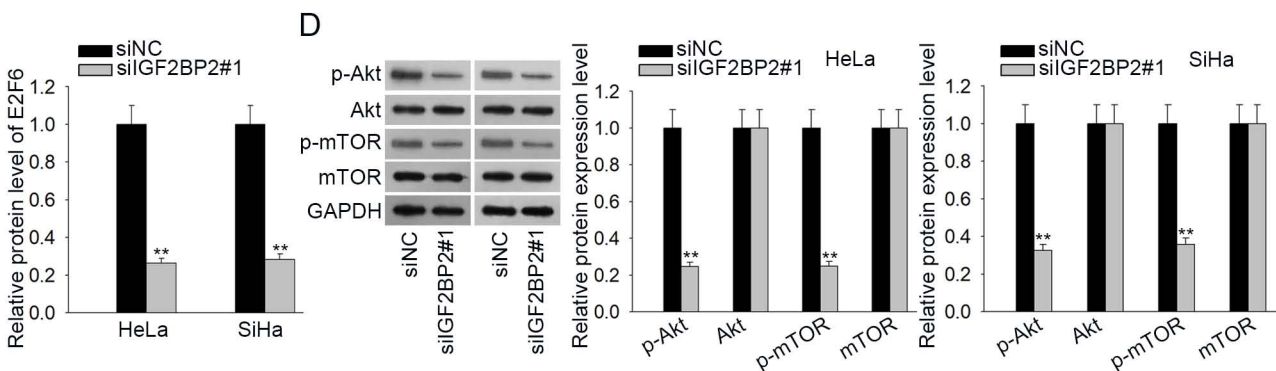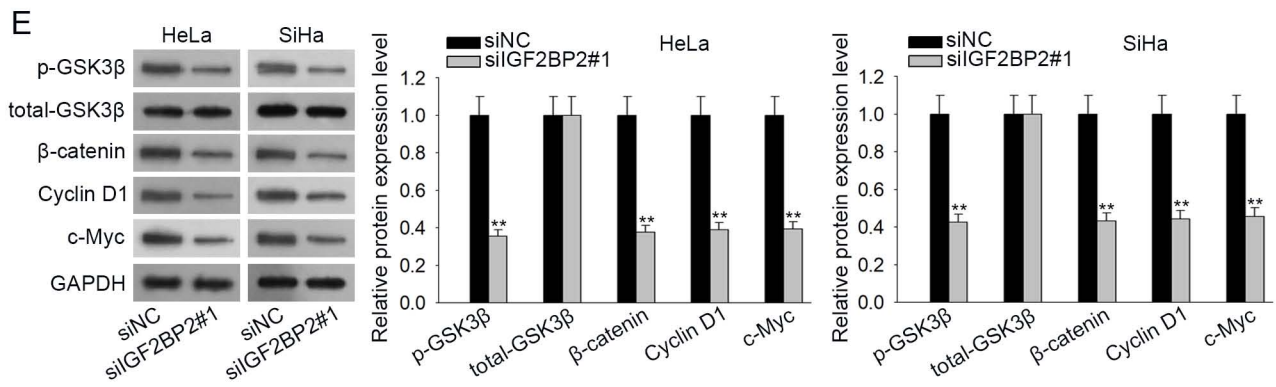

A

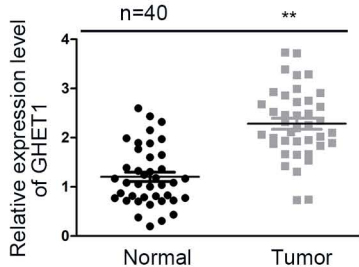

B

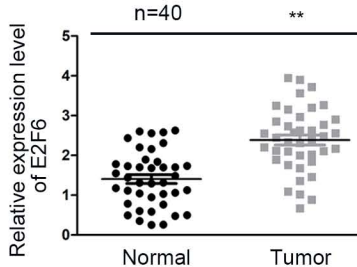

C

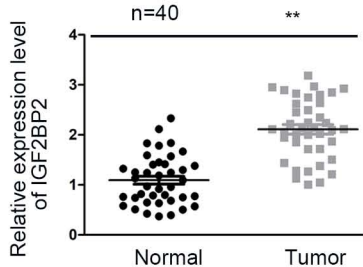

Supplement: Supplementary Figures S1-S2 [file BSR-2019-1265_supp.pdf]
